# Fusion proteins towards fungi and bacteria in plant protection

**DOI:** 10.1099/mic.0.000592

**Published:** 2017-12-14

**Authors:** Ana Margarida Pinheiro, Alexandra Carreira, Ricardo B. Ferreira, Sara Monteiro

**Affiliations:** ^1^​ LEAF – Linking Landscape, Environment, Agriculture and Food Instituto Superior de Agronomia, Universidade de Lisboa, 1349-017 Lisboa, Portugal; ^2^​ CEV, SA, Parque Industrial de Cantanhede/Biocant-Park, lote 120, 3060-197 Cantanhede, Portugal

**Keywords:** biopesticides, biotechnology, antimicrobial peptides, Blad-containing oligomer, pest management

## Abstract

In agriculture, although fungi are considered the foremost problem, infections by bacteria also cause significant economical losses. The presence of different diseases in crops often leads to a misuse of the proper therapeutic, or the combination of different diseases forces the use of more than one pesticide. This work concerns the development of a ‘super-Blad’: a chimeric protein consisting of Blad polypeptide, the active ingredient of a biological fungicide already on the market, and two selected peptides, SP10-5 and Sub5, proven to possess biological potential as antibacterial agents. The resulting chimeric protein obtained from the fusion of Blad with SP10-5 not only maintained strong antibacterial activity, especially against *Xanthomonas* spp. and *Pseudomonas syringae*, but was also able to retain the ability to inhibit the growth of both yeast and filamentous fungi. However, the antibacterial activity of Sub5 was considerably diminished when fused with Blad, which seems to indicate that not all fusion proteins behave equally. These newly designed drugs can be considered promising compounds for use in plant protection. A deeper and focused development of an appropriate formulation may result in a potent biopesticide that can replace, *per se,* two conventional chemistries with less impact on the environment.

## Introduction

The quality and yield of crop production have been adversely affected by a large variety of pests, including bacteria, fungi, weeds and insects [1]. Despite the undeniable role posed by fungi and viruses as the most important plant pathogens, bacterial infections are increasing and becoming more severe [2], making them a major threat to agriculture due to the lack of suitable agrochemicals and the absence of resistance and/or immunity in host plants. Moreover, they are usually spread undetected as contaminants or asymptomatic (latent) infections in plant propagation materials [3]. Another problem is common error when identifying a plant disease. Manual identification is a somewhat subjective task and laboratory analyses are often time consuming, leading to an unacceptable lag between observation and identification of the disease. Besides, the symptoms of some diseases are highly heterogeneous, making them difficult to identify. Moreover, the simultaneous presence of different diseases may exhibit a combination of symptoms quite different from the each disease *per se* [4]. As a whole, these factors often lead to a misinterpretation of the disease which ultimately leads to a misuse of a proper therapeutic agent, or the combination of different diseases forces the use of more than one pesticide.

The abuse and incorrect use of agrochemicals has resulted in pesticide resistance, pest resurgence, outbreaks of secondary pests, and pesticide residues in the produce, soil, air and water [5]. Biopesticides are becoming more appealing because of their advantages in terms of effectiveness, environmental safety, specificity, biodegradability and suitability in integrated pest management (IPM) programmes. Thus, biopesticides are promising alternatives in the management of environmental pollutions. The use of a new generation of microbial biopesticides has registered a steady annual increase of 10 %, and more than 225 microbial biopesticides are registered in 30 Organization for Economic Co-operation and Development (OECD) countries [6]. Nevertheless, biopesticides are still a young and evolving science where in-depth research is still needed, from production and formulation to delivery and commercialization of these products.

For the past few years, antimicrobial peptides have emerged as promising therapeutic agents in both medicine and agriculture due to their relatively small size (ease of synthesis), their fastness and efficiency, their antimicrobial activity against a wide range of pathogens and their low toxicity for vertebrate cells [7, 8]. However, some studies have demonstrated that the simultaneous use of more than one antimicrobial peptide is usually a better approach toward increasing its effectiveness in plant protection or for pharmaceutical uses. To achieve this, a simple strategy is to fuse two protein sequences with the same specialized [9], or complementary, functions [10, 11], originating a novel protein called a fusion, or chimeric, protein [12].

Blad is the active ingredient of a biological fungicide already on the market and discovered by our team. It is a 20.4 kDa polypeptide, being the major subunit of a 210 kDa glyco-oligomer, termed Blad-containing oligomer (BCO), which accumulates in *Lupinus albus* cotyledons between days 4 and 12 after the onset of germination. The characteristics that makes the BCO a unique, versatile and multi-functional protein [13], as well as its powerful and broad-spectrum antifungal activity towards both plant and human pathogens, have been fully discussed [13, 14]. Its mode of action was also recently described as a multi-site fungicide that disturbs microbial cell homeostasis leading, ultimately, to cell death [15].

In this work, we report the development of a ‘super-Blad’ – a fusion gene consisting of the gene that codes for Blad and genes that code for selected peptides proven to possess biological potential as antibacterial agents. SP10-5 is a 12-amino acid peptide experimentally designed by Zeitler and colleagues, using the scorpion-derived antimicrobial peptide IsCT and the frog-derived peptide mangainin II as templates [16]. SP10-5 was tested against several bacteria and presented promising results [16]. Sub5 is a 12-amino acid cationic peptide synthesized from a linearized variant of the bovine peptide bactenecin, Bac2A, that acts on the external membrane of Gram-negative bacteria [17]. Sub5 presents not only antibacterial activity against an array of both Gram-positive and -negative bacteria, but also antifungal activity against *Candida albicans* [17, 18].

The current investigation is in line with the new approach towards producing novel fusion proteins with improved antimicrobial performance, which may represent the future of technological development of biopesticides. Here we report the fusion of two proteins with distinct antimicrobial properties, one fungicidal and the other bactericidal, creating a new ‘super-molecule’ that enables simultaneous treatment of fungal and bacterial infections in plants.

## Methods

### Strains and media

DH5α-competent *Escherichia coli* cells were used for all routine cloning experiments. For recombinant protein overexpression, C41 (DE3) *E. coli* strain was used, cultivated in TB (12 g l^−1^ tryptone, 24 g l^−1^ yeast extract, 9.4 g l^−1^ K_2_HPO_4_, 2.2 g l^−1^ KH_2_PO_4_ and 4 ml l^−1^ glycerol) medium.

Strains of *E. coli*, *Salmonella thypimurium*, *Pseudomonas aeruginosa* and *Staphylococcus aureus* were provided courtesy of Instituto Superior de Agronomia (ISA-UL). Strains of *Xanthomonas arboricola* pv. *pruni* (NCPPB 2878 and NCPPB 3744), *X. versicatoria* (NCPPB 3801 and NCPPB 3954) and *Pseudomonas syringae* pv. *tomato* (NCPPB 3645 and NCPPB 4369) were purchased from the National Collection of Plant Pathogenic Bacteria (NCPPB, UK). All strains were grown at 34 °C for 24 h in tryptone soy agar (TSA) medium [3 % (w/v) tryptone soy broth, 1.5 % (w/v) agar]. For antibacterial susceptibility testing, bacteria were grown in Mueller–Hinton medium.

*C. albicans* var. *albicans* (CBS 562) {CBS: Centraalbureau voor Schimmelcultures} and *C. glabrata* (a kind gift from of the Institute of Microbiology, Faculty of Medicine of the University of Coimbra [19]) were grown at 34 °C for 24 h in glucose yeast peptone (GYP) medium [1 % (w/v) peptone, 0.5 % (w/v) yeast extract, 2 % (w/v) glucose and 1.5 % (w/v) agar].

*Botrytis cinearea* was isolated in our laboratory (from tomato) and was grown on Sabouraud dextrose agar for 7 days at 25 °C. For antifungal susceptibility testing, the medium used was PDB [2.4 % (w/v) potato dextrose broth], buffered at pH 7.5.

### Plant material

*Lupinus albus* L. seeds were purchased from Inveja SAS (France) and were germinated as described by Pinheiro and colleagues [15].

### Production of BCO

Blad-containing oligomer (BCO) was extracted and purified from 8-day-old cotyledons as previously described [20], and stored lyophilized at room temperature.

### Design of synthetic genes and cloning procedures

Synthetic genes were purchased from GeneScript, Hong Kong and cloned into pCoofy plasmids, a bacterial expression vector containing N-terminal 6x His and MBP tags [21]. General cloning procedures were performed as described by Scholz and colleagues [22]. The correct nucleotide sequence of the inserts in all the constructed plasmids was checked by DNA sequencing.

### Expression of the fusion proteins

Cells harbouring plasmids encoding the different fusion proteins were grown overnight at 37 °C with shaking, in TB medium supplemented with kanamycin. For the initial expression screening, 20 µl of the preculture were inoculated into 2 ml of fresh media on a 24-well plate. For production upscaling, 10 ml of the preculture were used to inoculate 1 l of fresh media. Inoculates were grown at different temperatures (20, 25, 30 and 37 °C) until OD_600 nm_ of 0.4 and then induced with three different concentrations of IPTG (isopropyl β-D-1-thiogalactopyranoside) (0.1, 0.5 and 1 mM). The cells were pelleted after 6 h by centrifugation at 4000 ***g***, 4 °C, for 20 min.

### Fusion protein purification

Cell pellets were resuspended in lysis buffer (50 mM Tris-HCl pH 8.0, 150 mM NaCl and 0.25 mg ml^−1^ lysozyme), submitted to a freeze/thaw cycle and then incubated with DNase (2.5 µg ml^−1^) and MgCl_2_ (5 mM), thus originating the total fraction. The soluble and insoluble fractions were separated by centrifugation (3500 ***g***, 10 min, 4 °C).

For purification purposes, a MBPTrap HP column (GE Healthcare) was used, previously equilibrated with 20 mM Tris-HCl at pH 8.0, 2 mM ethylenediamine tetraacetic acid (EDTA), 150 mM NaCl and 10 % (v/v) glycerol, and eluted with 10 mM maltose in binding buffer.

### Solubilization and re-folding of inclusion bodies

The solubilization and re-folding of inclusion bodies was performed as described by Singh and colleagues [22]. Briefly, the insoluble fraction resulting from the expression of the fusion proteins was resuspended in wash buffer A (50 mM Tris-HCl pH 8.5, 5 mM EDTA, 1 mM phenylmethylsulfonyl fluoride (PMSF)) and centrifuged at 20 000 ***g*** for 20 min at 4 °C. The pellet was resuspended in wash buffer B (50 mM Tris-HCl pH 8.5) and centrifuged again under the same conditions. The final pellet was resuspended in milliQ water and kept frozen at −80 °C until used. For solubilization purposes, 5 mg of inclusion bodies were suspended in solubilization buffer (50 mM Tris-HCl pH 8.5, 5 mM EDTA, 1 mM PMSF, 8 M urea), mixed by vortexing and incubated at room temperature for 1 h. The solubilized proteins were centrifuged at 20 000 ***g*** for 30 min at 4 °C. For the re-folding, 1 ml of the supernatant was added in small amounts in regular intervals to 9 ml of freshly cooled re-folding buffer (50 mM Tris-HCl pH 7.5, 1 mM EDTA, 10 % (w/v), 1 mM PMSF). The re-folded sample was kept at 4 °C for 6 h and then filtered through a 0.45 µM polyvinylidene fluoride (PVDF) filter to remove protein aggregates.

### Liquid chromatography tandem-mass spectrometry (LC-MS/MS) analysis

#### In-gel digestion

The gel bands were sliced into small pieces, de-stained and incubated overnight with trypsin for protein digestion. Following digestion, peptides were extracted from the gel using three different solutions of increasing percentages of organic solvent [water/acetonitrile with 1 % (v/v) formic acid]. Peptides were resuspended in 30 µl of a solution containing 2 % (v/v) acetonitrile and 0.1 % (v/v) formic acid and analysed by LC-MS/MS.

#### LC-MS/MS analysis

Samples were analysed on an AB Sciex 5600 TripleTOF (ABSciex) in information-dependent acquisition (IDA) mode. Peptides were fractionated by liquid chromatography (nanoLC Ultra 2D, Eksigent) on a MicroLC column ChromXPTM C18CL reverse-phase column (300 µm ID×15 cm length, 3 µm particles, 120 Å pore size, Eksigent) at 5 µl min^−1^ and eluted into the mass spectrometer with an acetonitrile gradient in 0.1 % FA (2 –30 % ACN, in a linear gradient for 30 min), using an electrospray ionization source (DuoSprayTM Source, ABSciex) with a 50 µm internal diameter (ID) stainless steel emitter (New Objective). For information-dependent acquisition (IDA) experiments, the mass spectrometer was set to scanning full spectra (350–1250 m/z) for 250 ms, followed by up to 80 MS/MS scans (100–1500 m/z from a dynamic accumulation time – minimum 30 ms for precursor above the intensity threshold of 1000 – in order to maintain a cycle time of 2.7 s). Candidate ions with a charge state between +2 and +5 and counts above a minimum threshold of 10 counts s^–1^ were isolated for fragmentation, and one MS/MS spectrum was collected before adding those ions to the exclusion list for 15 s (mass spectrometer operated by Analyst TF 1.6, ABSciex). Rolling collision was used with a collision energy spread of 5.

#### Protein identification

Protein identification was performed using Protein Pilot software (v 5.1, ABSciex) with the following parameters: search against the theoretical sequences of the recombinant proteins and against the uniprot database from June 2016, with acrylamide alkylation and trypsin digestion. Positive identification was considered for proteins that met the 1.3 unused score value and 95 % peptide confidence filtering.

### Yeast and bacteria inhibition tests

The susceptibility tests were performed according to the CLSI – Clinical and Laboratory Standards Institute, guidelines M27-A2, M31-A2 and M38-A2, for yeasts, bacteria and filamentous fungi, respectively [23–25] with minor adjustments.

Bacteria were grown on TSYA medium overnight at 34 °C. The inoculum suspension was then prepared in 5 ml of sterile 0.9 % (w/v) saline solution (NaCl) and cell density was adjusted with a spectrophotometer in order to obtain a concentration of 1×10^8^ cells ml^−1^. The final inoculum suspension was made by a 1 : 100 dilution with double-strength Mueller–Hinton medium, in order to achieve a concentration of 1×10^6^ cells ml^−1^. The inoculum size was confirmed by enumeration of c.f.u. on TSYA plates.

The susceptibility tests on yeasts and filamentous fungi were performed as previously described [13, 14].

In all cases, each well of the microplate contained 100 µl of the inocula and 100 µl of the diluted drug solution (twofold).

The microplates were incubated at 34 and 25 °C for yeasts and filamentous fungi, respectively, and examined after 72 h. Bacteria were incubated at 30 °C and the results were taken after 24–72 h, according to their growth rate. The minimum inhibitory concentration (MIC) endpoints were recorded visually as the lowest drug concentration that showed absence of growth. All assays were performed in triplicate.

### Electrophoresis and immunoblotting

One-dimensional, sodium dodecyl sulphate-polyacrylamide gel electrophoresis (SDS-PAGE) and Western blotting were performed as previously described [20]. For the dot-blot, 2 µl of each sample were applied to a nitrocellulose membrane (Roche, Mannheim, Germany). Membranes were blocked with TBST (50 mM Tris, 150 mM NaCl, 0.1 % (v/v) Tween 20) supplemented with 5 % (w/v) skim milk, for 1 h at room temperature. Membranes were incubated with the antibody anti-His (Genescript) (1 : 2000) in buffer TBSTM [TBST, 0.5 % (w/v) skim milk] (1 : 10 000) for 1 h, at room temperature and, after washing, incubated with the antibody anti-mouse (1 : 10 000) (GE Healthcare, Buckinghamshire, UK). The detection was performed by degradation of the ECF substrate (GE Healthcare) and detection on a FX apparatus (Bio-Rad, Hercules, CA).

## Results and Discussion

### Production, expression and purification of recombinant peptide-fusion proteins

**Fig. 1.**
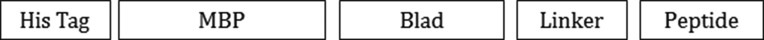
Production of the recombinant peptide-fusion proteins in *E.coli*. Design of both peptide-fusion proteins.

The genes that code for both SP10-5 and Sub5 peptides were fused with the Blad gene to construct the new multifactorial peptide-fusion proteins. The fusion of multiple proteins has already been extensively discussed for biotechnological applications [26–32]. Both genes were joined, separately, to the C-terminal portion of the Blad gene using a 5-amino acid flexible linker (Gly-Gly-Ser-Gly-Gly). Flexible linkers are usually applied when the joined domains require a certain degree of movement or interaction. The small size of these amino acids provides flexibility, and allows for mobility of the connecting functional domains [33]. The performance of the newly synthesized fusion protein depends largely on the peptide liner that is inserted between the moieties [34–38]. Considering the results obtained in a previous study where recombinant Blad was successfully produced in a soluble form in *E. coli* using MBP (maltose-binding protein) as a solubility enhancement partner (unpublished work), the MBP gene was also added to the construction in the N-terminal end of Blad. MBP has been successfully used as a solubility-enhancing partner, with excellent results [39–41] and the additional benefit that it can also be used as an affinity tag for purification [42, 43]. The design of the peptide-fusion protein construction is shown in Fig. 1 and the final amino acid sequences are shown in Fig. S1 (available in the online version of this article). MBP has a molecular weight of 40.2 kDa, Blad is 20.4 kDa and SP10-5 and Sub5 are 1.5 and 1.7 kDa, respectively. Therefore, the molecular weight of both peptide-fusion proteins was approximately 60 kDa.

**Fig. 2.**
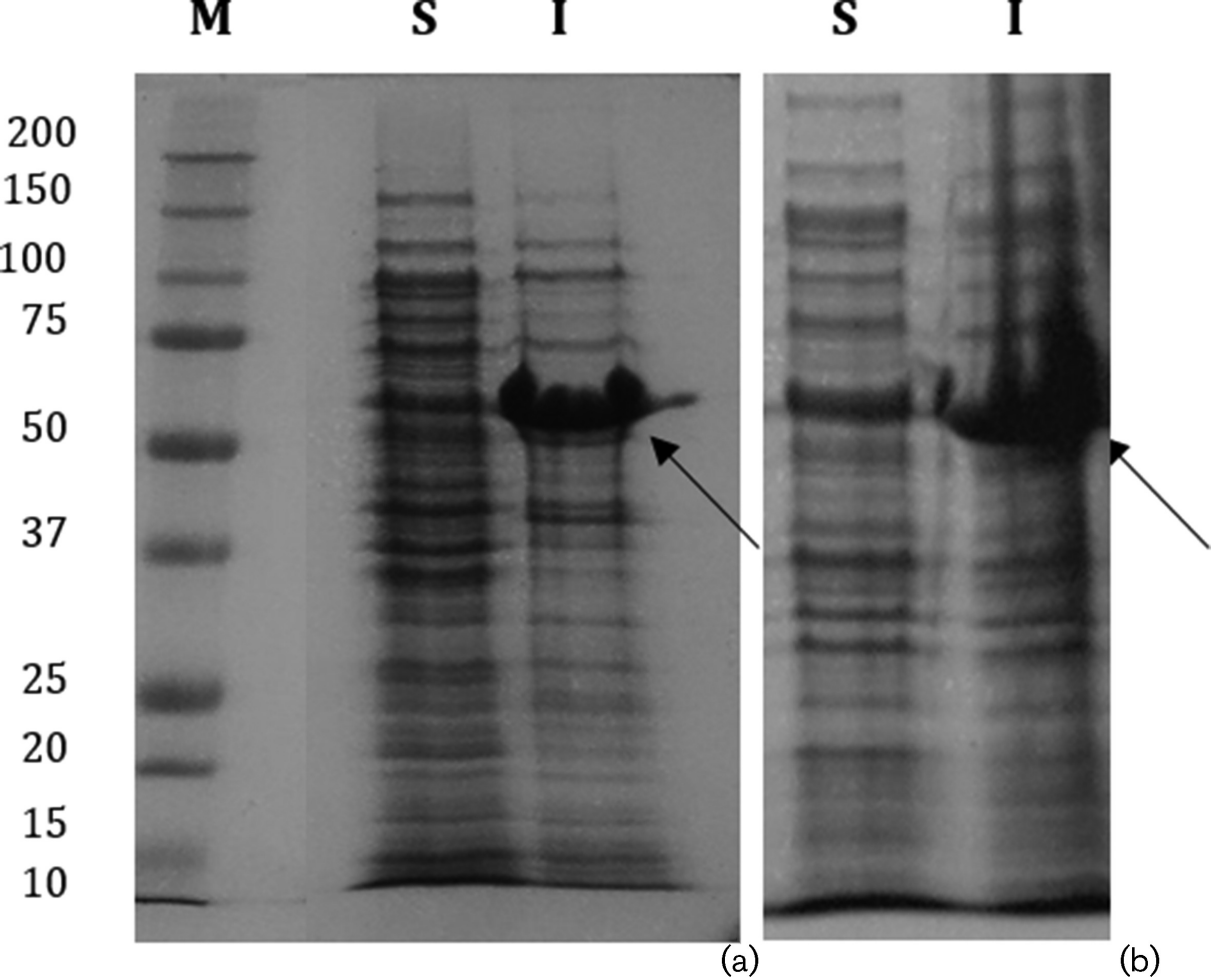
SDS-PAGE analysis of both soluble (S) and the insoluble (I) fractions resulting from the expression of fusions His_6_MBPBladSP10-5 (a) and His_6_MBPBladSub5 (b). Molecular masses of standards are indicated in kDa.

Both synthetic genes that code for the new peptide-fusion proteins, hereby termed His_6_MBPBladSP10-5 and His_6_MBPBladSub5, were cloned into pCoofy plasmids, a bacterial expression vector for parallel sequence and ligation independent cloning (SLIC) containing N-terminal 6x His and MBP tags [21]. After induction of overexpression of the genes that code for both peptide-fusion proteins, cells were collected by centrifugation and analysed by both SDS-PAGE and immunoblot, using an anti-Histag antibody, which demonstrated that the genes that code for both peptide-fusion proteins were correctly over-expressed and at the expected molecular weight (~60 kDa) (data not shown). To assess the solubility of both peptide-fusion proteins, both soluble and insoluble fractions were loaded onto a 12 % (w/v) SDS-polyacrilamide gel. Fig. 2 shows that both peptide-fusion proteins were present mainly in the insoluble fraction under the growth conditions tested.

**Fig. 3.**
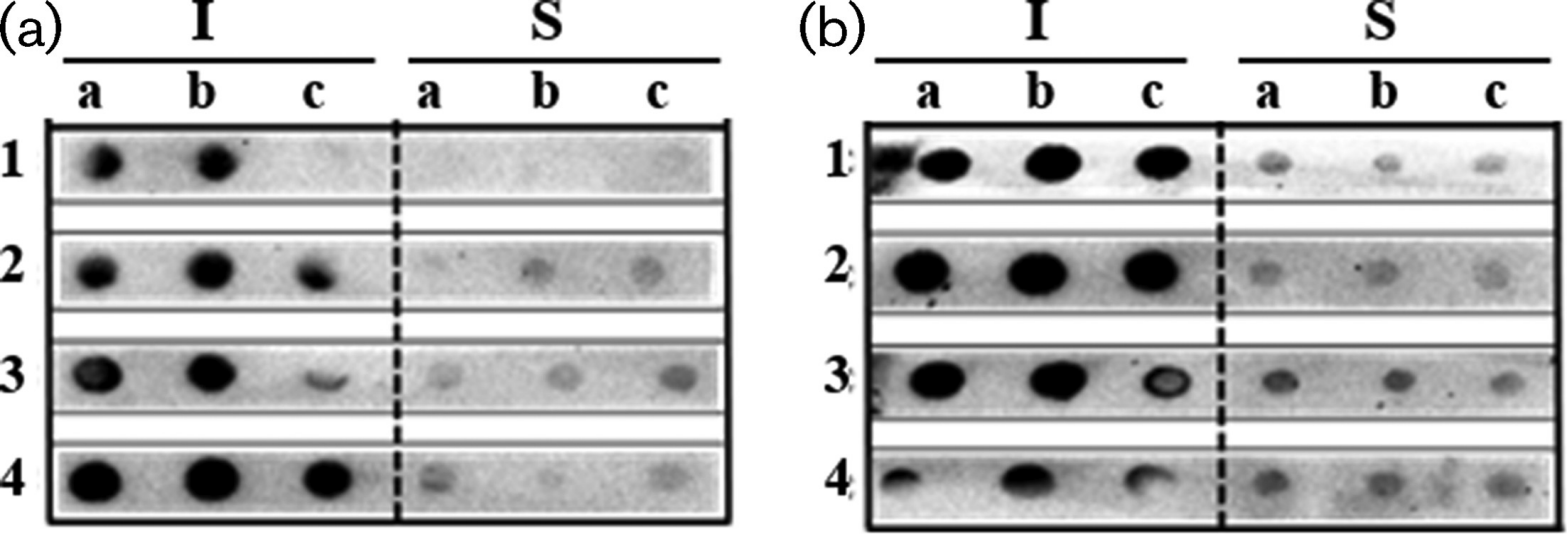
Dot-blot analysis of both the soluble (S) and insoluble (I) fractions resulting from the expression of fusions His_6_MBPBladSP10-5 (a) and His_6_MBPBladSub5 (b) at four different temperatures (1–20 °C; 2–25 °C; 3–30 °C and 4–37 °C) with three IPTG concentrations (a – 1 mM, b – 0.5 mM and c – 0.1 mM).

Another attempt was made to overcome the insolubility issues. Several expression conditions were tested, including a combination of four different temperatures (20, 25, 30 and 37 °C) with three IPTG concentrations (0.1, 0.5 and 1 mM). After expression under these conditions, both soluble and insoluble fractions were spotted directly onto a nitrocellulose membrane for dot-blot analysis, using an anti-histag antibody (Fig. 3). The results obtained revealed that the majority of the signal is in the insoluble fraction, despite the temperature or the IPTG concentration tested. The strong signal observed in the insoluble fraction of both peptide-fusion proteins is representative of high levels of expression. However, the fusion proteins were synthesized as inclusion bodies, meaning that none of the constructs led to significant accumulation of the fusion protein in a soluble form. Despite the accumulated evidence indicating that MBP is a very promising solubility-enhancing partner [39–41], no affinity tag is universally ideal and many have the same downside of yielding different performances with different partner proteins [43, 44]. Fusing an insoluble protein to a solubility-enhancing partner does not guarantee a soluble fusion protein [39].

There are multiple factors that contribute towards the formation of protein aggregates during heterologous expression in *E. coli*. Under normal circumstances, one protein chain is released from the ribosome of *E.coli* every 35 s and the macromolecule concentration can reach 300–400 mg ml^−1.^ Under these conditions the correct folding of a protein can be considered an extraordinary event [45]. In addition, the newly synthesized recombinant protein is being synthesized in an environment that may be quite different from that of its original source in terms of pH, osmolarity, redox potential, co-factors and folding mechanisms [46]. Finally, it may also be the case that insolubility is an intrinsic property of a particular protein [41]. Either way, if a given protein fails to rapidly achieve its native conformation there are two possible consequences: partial or complete deposition into inclusion bodies [45]. Tight control of a variety of parameters, including temperature of expression, expression rate and inducer concentration, might help to reduce the formation of aggregates [37, 43]. In many cases, the overexpression of genes in *E.coli* remains an unsolved problem as the proteins continue to be synthesized as inclusion bodies.

**Fig. 4.**
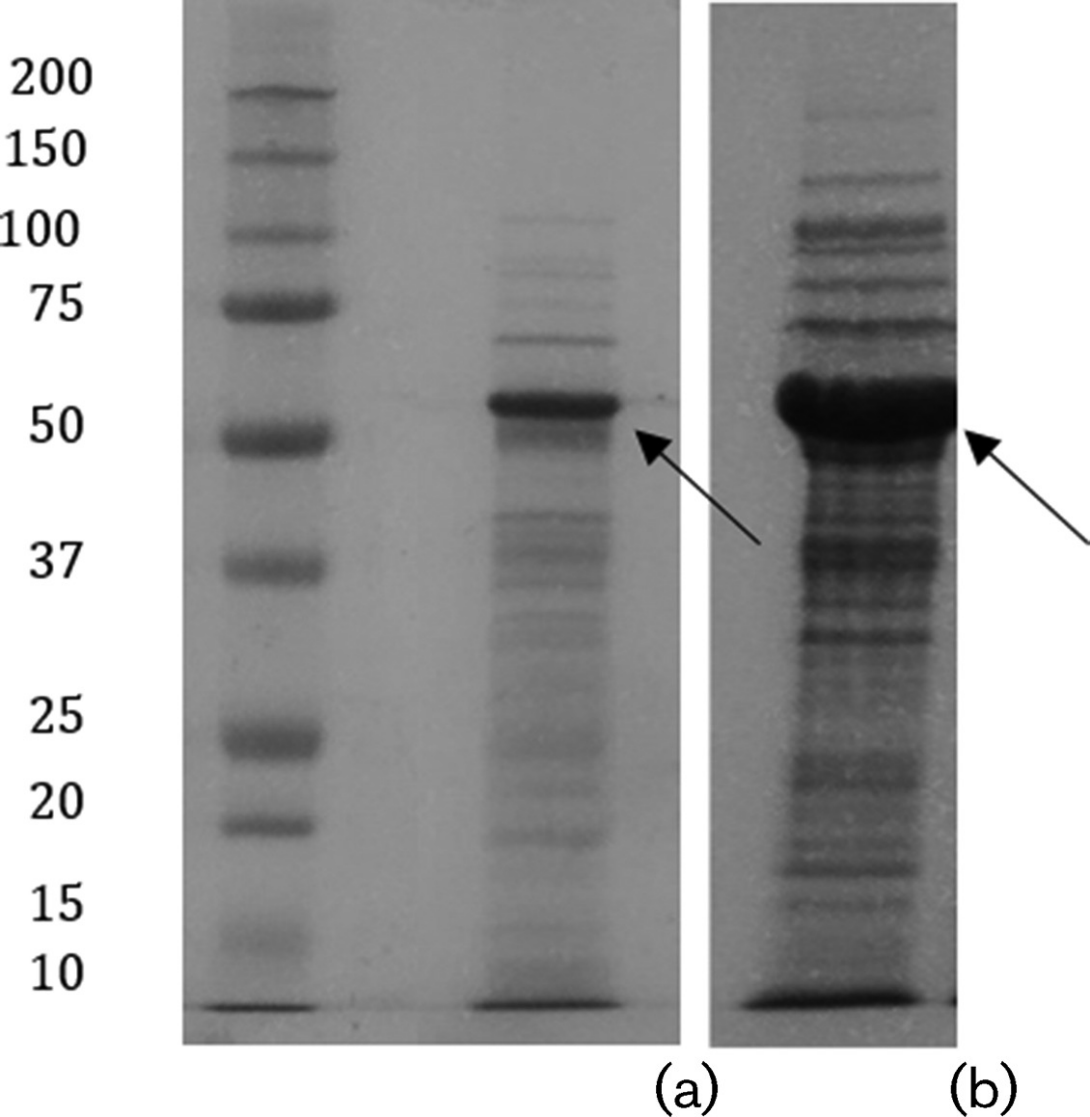
SDS-PAGE analysis of the solubilized inclusion bodies of His_6_MBPBladSP10-5 (a) and His_6_MBPBladSub5 (b). Molecular masses of standards are indicated in kDa.

Considering the impossibility of produci both fusion proteins in a soluble form, even with the MBP in the construction as a solubility-enhancing tag, the next step was to solubilize and re-fold the inclusion bodies, as described in Methods. The solubilized peptide-fusion proteins were analysed by SDS-PAGE (Fig. 4) and the results obtained revealed that, despite both His_6_MBPBladSP-10 and His_6_MBPBladSub5 being synthesized as inclusion bodies and thus only soluble in denaturant buffers, it is possible to re-fold them by slow dilution in a renaturing buffer. Moreover, Fig. 4 shows that compared to His_6_MBPBladSP10-5, His_6_MBPBladSub5 was recovered at a higher yield considering the thickness of the respective bands.

The next step was to purify the peptide-fusion proteins. As mentioned above, MBP not only is a powerful solubility-enhancing tag that often helps folding of fusion proteins [47], but also can be used as a detection and a purification tag [48]. Considering the addition of the MBP into the constructs, the purification was performed by affinity chromatography using a MBPTrap HP, a column prepacked with dextrin, a resin for which MBP has high affinity. The solubilized peptide-fusion proteins were applied to the prepacked column, washed and eluted with 10 mM maltose in binding buffer and the eluted fractions of both peptide-fusion proteins were analysed by SDS-PAGE (Fig. S2). Analysis of both the chromatogram and SDS-PAGE revealed that both fusion proteins were purified to a high level, which is in accordance with previous studies claiming that purification by capture affinity step on amylose columns resulted in a protein that is often 70–90 % pure [48, 49].

For the confirmation of the fusion proteins in this study, the corresponding bands were individually cut from the gel and analysed by LC-MS/MS. The results obtained from mass spectrometry analysis were somewhat inconclusive. Taking into consideration the specific regions for the peptides SP10-5 and Sub-5 (‘LRIIKKILKKLI’ and ‘RRWKIVVIRWRR’, respectively), it was difficult to obtain a good tryptic peptide for protein identification (trypsin cleaves after lysines and arginines) and thus to be able to identify the recombinant peptides. Both Blad and MBP were positively identified with a 95 % confidence level.

Despite the high purity level obtained when purifying both His_6_MBPBladSP-10 and His_6_MBPBladSub5 fusion proteins, the yields obtained, considering the need to re-fold the inclusion bodies by diluting them into a re-folding buffer, were relatively low. In fact, though formation of inclusion bodies renders protein purification easier, there is no guarantee that *in vitro* re-folding will generate large amounts of biologically active products [50].

### *In vitro* antimicrobial activity of fusion proteins

In a new set of experiments, the antimicrobial activity of the fusion proteins His_6_MBPBladSP10-5 and His_6_MBPBladSub5 was evaluated and compared to that of each individual active compound (SP10-5, Sub-5 and BCO). The results shown in Table 1 reveal that BCO has a potent antifungal activity against both yeasts and filamentous fungi, as expected [13, 14]. However, it was also demonstrated that BCO has some antibacterial activity, although not strongly and not against all species. By fusing Blad, the active ingredient of BCO, to a molecule proven to possess biological potential as an antibacterial agent [16, 17], its spectrum of action should, theoretically, be enhanced.

**Table 1.** *In vitro* susceptibility of various species of bacteria, yeasts and filamentous fungi to the fusion proteins His_6_MBPBladSP10-5 and His_6_MBPBladSub5 and to SP10-5, Sub5 and BCO, as determined by MIC (minimum inhibitory concentration)

Species* (no. of strains)	MIC range (µM)				
	SP10-5	His_6_MBPBladSP10-5	Sub5	His_6_MBPBladSub5	BCO
*X. arboricola* (2)	1.3–5.3	0.065	0.6–2.4	1.032–0.516	1.2–2.4
*X. vesicatoria* (2)	1.3–5.3	0.065	0.6–1.2	1.032	2.4
*E. coli* (1)	>13	0.516	4.7	na	na
*S. typhimurium* (1)	13	0.258	9.4	na	na
*P. aeruginosa* (1)	>13	0.516	9.4	na	na
*P. syringae* (2)	1.3	0.065	1.2	1.032	0.6–1.2
*S. aureus* (1)	13	1.032	9.4	1.032	2.4
*C. albicans* (1)	13	0.032	37.6	0.065	0.074
*C. glabrata* (1)	42.7	0.065	>37.6	0.032	0.074
*B. cinerea* (1)	42.7	0.065	37.6	0.065	0.149

na, No activity within the range of concentrations tested.

* *B. Botrytis*, *C. Candida, E. Escherichia*, *P. Pseudomonas*, *S. Salmonella*, *X. Xanthomonas.*

Bacterial susceptibility to SP10-5 alone was variable, but its potent effect towards *Xanthomonas* spp. and *P. syringae* was confirmed [16]. Although Sub5 is described as having considerable antibacterial activity against several species [17, 18], in our study MICs were somewhat higher than expected, but its efficacy and wide spectrum range were confirmed. When fused, the activity of SP10-5 (His_6_MBPBladSP10-5) was highly enhanced for all bacteria, becoming particularly efficient against *Xanthomonas* spp. and *P. syringae*. The most extraordinary results were obtained for *E. coli* and *P. aeruginosa* because Blad and SP10-5 alone were inactive against these species, but their fusion inhibited bacterial growth. The same occurred with Sub5 for these species as well as for *S. typhimurium*. Furthermore, the antibacterial activity of the fusion protein His_6_MBPBladSub5 towards the remaining species was always lower than the corresponding activity of the individual compounds alone, suggesting a synergistic effect between these molecules. Comparing the antifungal activity of both fusion proteins to that of BCO alone, the construction was able to retain the ability to inhibit the growth of both yeasts and filamentous fungi. This was corroborated by the MIC values of Sub-5 and SP10-5 alone for these strains, which were much higher than those observed when fused to Blad but similar to the values of BCO alone, thus confirming that the antifungal activity of the hybrid proteins derives exclusively from Blad. The antimicrobial activity of the isolated MBP was tested and revealed no influence on the results (data not shown).

Fusion proteins are now extensively used in the biomedical field as an important tool to detect and purify antibodies [51], and to design and produce bifunctional enzymes [52]. In this work, we designed and investigated the antimicrobial activity of the fusion proteins His_6_MBPBladSP10-5 and His_6_MBPBladSub5 against important plant pathogens. Both fusions were constructed with the peptide genes fused to the C-terminal portion of Blad using a 5-amino acid flexible linker (oligopeptide) that is widely used to construct functional fusion proteins. As the name implies, flexible linkers are composed of flexible amino acid residues such as glycine, serine and proline, which allows contiguous proteins to move independently [10].

It is difficult to express antimicrobial proteins in bacteria, simply because the active antimicrobial proteins will kill the host. One possible approach is to force the expression of the target protein as an inclusion body [53, 54] which, unwittingly, was the case here from the beginning. Thus, we were able to express a bactericidal peptide in *E. coli* without compromising its viability. However, it is well known that even after successful expression of antimicrobial fusion proteins, they constantly present diminished or absent antimicrobial activity [9]. In addition, our results seem to indicate that not all fusion proteins behave equally; when fusing genes that code for two different proteins, one can eventually be produced with impaired bioactivity. Despite the idea that the choice of the component proteins is based only on the desired functions of the fusion protein and that, in most cases, is relatively straightforward [33], the design of fusion proteins with desired and predictable behaviour remains a daunting challenge [32].

In this work, both constructions retained the ability to inhibit the growth of both yeasts and filamentous fungi and, most importantly, both showed increased antibacterial activity, probably due to a synergistic effect between Blad and SP10-5 and Sub5. Hongbiao *et al*. [9] and Lee *et al*. [10] had already successfully engineered hybrid proteins from two antibacterial peptides. However, the originated fusion proteins presented inferior antibacterial activity compared to the individual parental ones. In a similar study published recently by Kovalskaya *et al*. [11], a hybrid protein, named SAP, was also successfully produced but, in this case, it showed distinct and enhanced biological activities. The results indicate that the performance of the hybrid protein was better than or equal to that of the parental proteins. The work reported by Kovalskaya *et al*. [11] also demonstrated that the chimeric protein SAP produced in tobacco and potato plants had a plant-protective role against *Colletotrichum coccoides*, the casual fungal agent of potato anthracnose, and *Clavibacter michiganensis* subsp. *sepedonicus*, which is the bacterium that causes potato ring rot disease.

Although this proved to be a very interesting study, the ultimate purpose of the authors was to develop a genetic strategy to express the hybrid protein SAP as a self-cleavage protein in plant cells to allow the production of individual antimicrobial proteins and, in this way, increase plant pathogen resistance. Our work aimed at generating a new molecule to be used as an active ingredient for plant protection as a plant foliar biopesticide. For a new biopesticide to be of plant protection interest, high efficiency needs to be combined with low toxicity. Although the toxicological profile of the hybrid proteins was not assessed in this study, both the BCO and the individual peptides were previously recognized as compounds of low toxicity. In the study recently published by Pinheiro *et al*. [15], BCO showed no evidence of topical toxicity, genotoxicity and carcinogenicity towards mammalian cells after acute or short-term exposure. In addition, BCO safety as a plant protection product has been corroborated by the national regulatory entities of all countries where the product has already been approved. Zeitler *et al*. [16] showed that SP-10 peptide was highly active against a broad spectrum of bacteria, but showed low haemolytic activity and a very low phytotoxicity to plant protoplasts and therefore seemed to be well suited as a plant protection agent. Ebbensgaard *et al*. [18] reported that Sub-5 peptide had no minimal haemolytic activity, indicating that it might be safe to use at the concentrations needed to exert antibacterial activity. In conjunction with both the high efficacy and low toxicity profile, allied to a future deeper and more focused development of an appropriate formulation, this may result in a potent protein-based biopesticide for use in agriculture.

The results presented in this study are an important breakthrough and may represent a new approach in the design of new bi-functional antimicrobial peptides with major potential for use in medicine, food safety and agriculture. In conclusion, we designed, engineered and produced fusion proteins with antimicrobial activity against a range of human, spoilage and plant-pathogenic bacteria, yeasts and filamentous fungi.

## Supplementary Data

140 Supplementary File 1
