# Supplementary material for: Fusion proteins towards fungi and bacteria in plant protection
Source: Microbiology (Reading). 2017 Dec 14;164(1):11–9. doi: 10.1099/mic.0.000592 (PMC5892777; doi:10.1099/mic.0.000592)
Supplement: Supplementary File 1 [file mic-164-11-s001.pdf]

SP10-5

**MKMNKSLIVLCLSAGLLASAPGISLADVNYVPQNTSDAPAIPSAALQQ
LTWTPVDQSKTQTTQLATGGQQLNVPGISGPVAAYSVPANIGELTLTL
TSEVNKQTSVFAPNVLILDQNMTPSAFFPSSYFTYQEPGVMSADRLEG
VMRLTPALGQQKLYVLVFTTEKDLQQTTQLLDPAKAYAKGVGNSIPDI
PDPVARHTTDGLLKLKVKTNSSSSVLVGPLFGSSAPAPVTVGNTAAPA
VAAPAPAPVKKSEPMLNDTESYFNTAIKNAVAKGDVDKALKLLDEAE
RLGSTSARSTFISSVKGKG**RRQRNPYHFSSQRFQTLYKNRNGKIRVLERF
DQRTNRLENLQNYRIVEFQSKPNTLILPKHSDADYVLVVLNGRATITIVN
PDRRQAYNLEYGDALRIPAGSTSYILNPDDNQKLRVVKLAIPINNPGYFYD
FYPSSTKDQQSYFSGFSRNTLEATFNTRYEEIQRIILGNEDGGSGGLRIIKK
ILKKLI

A

Sub5

**MKMNKSLIVLCLSAGLLASAPGISLADVNYVPQNTSDAPAIPSAALQQ
LTWTPVDQSKTQTTQLATGGQQLNVPGISGPVAAYSVPANIGELTLTL
TSEVNKQTSVFAPNVLILDQNMTPSAFFPSSYFTYQEPGVMSADRLEG
VMRLTPALGQQKLYVLVFTTEKDLQQTTQLLDPAKAYAKGVGNSIPDI
PDPVARHTTDGLLKLKVKTNSSSSVLVGPLFGSSAPAPVTVGNTAAPA
VAAPAPAPVKKSEPMLNDTESYFNTAIKNAVAKGDVDKALKLLDEAE
RLGSTSARSTFISSVKGKG**RRQRNPYHFSSQRFQTLYKNRNGKIRVLERF
DQRTNRLENLQNYRIVEFQSKPNTLILPKHSDADYVLVVLNGRATITIVN
PDRRQAYNLEYGDALRIPAGSTSYILNPDDNQKLRVVKLAIPINNPGYFYD
FYPSSTKDQQSYFSGFSRNTLEATFNTRYEEIQRIILGNEDGGSGGRRWKI
VVIRWRR

B

Fig.S1. Amino acid sequences of the peptide-fusion proteins with SP10-5 **(A)** and Sub5 **(B).**

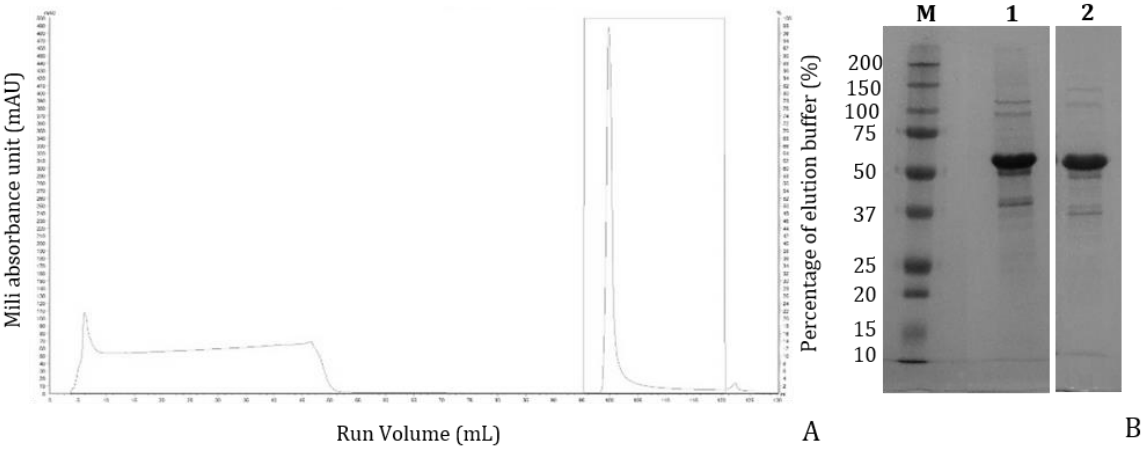

Fig. S2. Chromatogram obtained after purification of the His_6MBPBladSP-10 fusion protein by affinity chromatography using a MBPtrap column, eluted with 10 mM maltose in binding buffer (**A**) and SDS-PAGE analysis of the eluted fractions of the peptide-fusion protein His_6MBPBladSP10-5 and His_6MBPBladSub5 (lanes 1 and 2, respectively) (**B**). Molecular masses of standards are indicated in kDa.
